# Impact of Yoga in Transformation of Quality of Life of Alzheimer’s disease cases

**DOI:** 10.1002/alz.095088

**Published:** 2025-01-09

**Authors:** Meenakshi Kaushik

**Affiliations:** ^1^ All India Institute of Medical Sciences, New Delhi, Delhi, Delhi India

## Abstract

**Background:**

Alzheimer’s disease (AD) constitutes a formidable challenge, profoundly affecting the fabric of individuals' lives and intricately entwined with disturbances in their quality of life. This study embarks on a mission to unravel the transformative potential of a carefully crafted 12‐week yoga intervention, specifically tailored to address the multifaceted dimensions of quality of life. Our aim is to unravel the impact of a yoga intervention on their overall quality of life with AD and mild cognitively impairment subjects.

**Method:**

A case control yoga interventional study was conducted on 30 subjects (male‐18 and female‐12) were enrolled from the department of neurology and neurocognitive assessments were done in department of Anatomy, AIIMS, New Delhi, India. All the participants, aged >60 years were recruited. The 12‐week yoga intervention entailed daily hour‐long sessions over 6 days. The multifaceted evaluation encompassed the Geriatric Depression Scale (GDS) including the evaluation of 15 questions and the Montreal Cognitive Assessment (MoCA) scale including for overall quality of life assessment with measuring language, memory, attention, visuospatial, naming, delayed recall, abstraction, and orientation for quality‐of‐life of the AD subjects.

**Result:**

The AD subjects cohort exhibited statistically significant enhancements (P < 0.001) in quality‐of‐life scores (GDS & MoCA) pre and post Yoga intervention. GDS scores of 15 questions experienced a remarkable transition from pre‐yoga (8.36 ± 2.7) to post‐yoga (5.13 ± 3.0). While total MoCA scores ascended from pre yoga (18.65 ± 4.13) to post yoga (25.06 ± 6.3). MoCA scores of individual points pre yoga includes language (1.10 ± 0.2), memory (no points), attention (3.54 ± 1.8), visuospatial (4.0 ± 1.30), naming (2.16 ± 0.4), delayed recall (3.55 ± 0.25), abstraction (0.50 ± 0.0), and orientation (3.80 ± 0.18). MoCA scores of individual points post yoga includes language (2.16 ± 0.6), memory (no points), attention (4.60 ± 1.3), visuospatial (4.90 ± 2.50), naming (2.85 ± 0.75), delayed recall (4.85 ± 0.50), abstraction (1.40 ± 0.20), and orientation (4.30 ± 0.45).

**Conclusion:**

This discerning study illuminates the transformative potential of a 12‐week yoga program, showcasing significant enhancements in the quality of life of individuals grappling with mild to moderate Alzheimer’s disease.

